# Interaction between Mesenchymal Stem Cells and Intervertebral Disc Microenvironment: From Cell Therapy to Tissue Engineering

**DOI:** 10.1155/2019/2376172

**Published:** 2019-09-10

**Authors:** Gianluca Vadalà, Luca Ambrosio, Fabrizio Russo, Rocco Papalia, Vincenzo Denaro

**Affiliations:** Department of Orthopaedic and Trauma Surgery, Campus Bio-Medico University of Rome, Via Alvaro del Portillo 200, 00128 Rome, Italy

## Abstract

Low back pain (LBP) in one of the most disabling symptoms affecting nearly 80% of the population worldwide. Its primary cause seems to be intervertebral disc degeneration (IDD): a chronic and progressive process characterized by loss of viable cells and extracellular matrix (ECM) breakdown within the intervertebral disc (IVD) especially in its inner region, the nucleus pulposus (NP). Over the last decades, innovative biological treatments have been investigated in order to restore the original healthy IVD environment and achieve disc regeneration. Mesenchymal stem cells (MSCs) have been widely exploited in regenerative medicine for their capacity to be easily harvested and be able to differentiate along the osteogenic, chondrogenic, and adipogenic lineages and to secrete a wide range of trophic factors that promote tissue homeostasis along with immunomodulation and anti-inflammation. Several *in vitro* and preclinical studies have demonstrated that MSCs are able to acquire a NP cell-like phenotype and to synthesize structural components of the ECM as well as trophic and anti-inflammatory mediators that may support resident cell activity. However, due to its unique anatomical location and function, the IVD presents distinctive features: avascularity, hypoxia, low glucose concentration, low pH, hyperosmolarity, and mechanical loading. Such conditions establish a hostile microenvironment for both resident and exogenously administered cells, which limited the efficacy of intradiscal cell therapy in diverse investigations. This review is aimed at describing the characteristics of the healthy and degenerated IVD microenvironment and how such features influence both resident cells and MSC viability and biological activity. Furthermore, we focused on how recent research has tried to overcome the obstacles coming from the IVD microenvironment by developing innovative cell therapies and functionalized bioscaffolds.

## 1. Introduction

Low back pain (LBP) is one of the most common musculoskeletal symptoms; it is estimated that up to 84% of adults will experience LBP at least once in their life, while more than 25% report to have suffered from an episode of LBP in the previous three months [1]. The peak prevalence of LBP occurs between 45 and 64 of age and is slightly more frequent in women, who usually complain of a higher rate of recurrence [2]. In addition, LBP is a major cause of disability and loss of working capacity worldwide [3], resulting in an enormous socioeconomic burden that significantly impacts on patients' quality of life as well as on healthcare expenditure. Indeed, it has been estimated that LBP is the second most common cause of loss of productive time among adult workers, especially if female, older than 60 years of age, and exposed to hostile and unsafe working conditions [4].

Although being triggered by several different causes, LBP is mainly provoked by intervertebral disc degeneration (IDD) [5]. The intervertebral disc (IVD) is a complex structure located between the vertebrae which provides the spine with bending capacity and shock-absorbing properties while helping in distributing mechanical loads across vertebral segments [6]. With the onset of IDD, the IVD especially its inner portion, namely, the nucleus pulposus (NP), undergoes a progressive dehydration due to proteolytic cleavage of aggrecan together with a substantial reduction of resident cell viability [7]. This ultimately impairs IVD biomechanical properties subsequently leading to structural alterations and development of discogenic LBP, as well as more severe sequelae, including disc herniation, spinal instability, and stenosis with serious neurological consequences [8]. To date, there is no treatment—neither conservative nor surgical—able to arrest or at least slow down the degenerative process. For this reason, several efforts are being made in order to develop innovative approaches to repair or ideally regenerate IVD original morphofunctional features.

One of the most appealing and promising strategies is disc regeneration through the supplementation of the degenerated IVD with exogenous mesenchymal stem cells (MSCs) [9, 10]. MSCs are multipotent adult stem cells provided with the capacity to self-renew and to differentiate into several tissues, including bone, cartilage, muscle, and fat. In the last decades, MSCs have been widely employed in different areas of regenerative medicine with promising results, especially in the musculoskeletal field and also in IDD. A major advantage of MSC-based treatments is their high accessibility as they can be easily and safely isolated from the bone marrow and the adipose tissue [9]. MSCs are identified upon three criteria proposed by the International Society for Cellular Therapy: (1) adherence to plastic, (2) marker expression (CD105^+^, CD73^+^, CD90^+^, CD45^−^, CD34^−^, CD14^−^ or CD11b^−^, CD79a^−^ or CD19^−^, and HLA-DR^−^), and (3) the capacity to differentiate along the chondrogenic, osteogenic, and adipogenic lineages [11]. The underlying concept is to induce the differentiation of MSCs towards a NP cell phenotype and/or to stimulate resident NP cells *via* released growth factors. This may increase the synthesis of extracellular matrix (ECM) main components, so as to regenerate the IVD [12, 13]. In the last 20 years, several preclinical and clinical studies have been conducted to confirm such proof of concept. Despite the incredible heterogeneity among these investigations (different animal models, cell sources and number, injection routes, and association with biomaterials), overall results showed a substantial improvement of LBP and IVD structure, even if no *restitutio ad integrum* was ever documented [12]. In addition, several studies demonstrated that injected MSCs rapidly become undetectable due to premature death soon after implantation [14, 15], while studies associating MSCs with bioscaffolds resulted in higher cell viability and improved differentiation, possibly by providing exogenous MSCs with a proper microenvironment [16, 17]. This has generated the hypothesis that the hostile environment of the degenerated IVD may critically impact on MSC survival, metabolism, and differentiation thus limiting or even abolishing their regenerative effect [18]. In order to tackle such limitations, innovative tissue engineering approaches have been investigated so as to mimic the original IVD healthy microenvironment by combining cell therapy, bioscaffolds, and soluble factors.

This review is aimed at describing the characteristics of the IVD microenvironment in both physiological and degenerative conditions and how they affect both endogenous and exogenous cell viabilities, functionalities, and phenotypes. Furthermore, this paper will discuss how recent research efforts have developed innovative cell therapies and biomaterials to overcome such harsh asperities and to encourage disc regeneration.

## 2. The Intervertebral Disc in Physiological Conditions

The IVD consists of three specialized tissues: the cartilaginous end plate (CEP), connecting the disc with the adjacent vertebral bodies, the annulus fibrosus (AF) and the NP [19].

The CEP is about 0.6 mm thick in humans and resembles hyaline articular cartilage in structure [20]. It is fundamental for the nourishment of the AF and NP; indeed, nutrients to the IVD pass through the bone marrow cavities of the vertebral bodies and then to the capillaries branching through the CEP, so that they can diffuse to the inner AF and NP which are definitely avascular [21].

The AF constitutes the external part of the IVD and surrounds the NP. It is composed of 15-25 concentric lamellae mainly made of type I collagen fibers directed radially and perpendicularly [22] with an interlamellar matrix containing noncollagenous proteins (*e.g*., elastin), proteoglycans, and fibroblast-like cells [23].

The NP is a gelatinous tissue mainly constituted by type II collagen and proteoglycans, especially aggrecan, which establishes a high level of hydration and swelling pressure due to the large number of sulfated glycosaminoglycans (GAGs) present in the ECM, providing the NP with its unique biomechanical properties [24]. Additional proteoglycans contained in the NP include versican, decorin, biglycan, and fibromodulin, as well as significant amounts of elastin, laminin, and fibronectin [25]. Within the NP, chondrocyte-like round cells, also known as nucleopulpocytes (NPCy) [12], can be found; such cells are characterized by specific markers, including hypoxia-inducible factor (HIF) 1*α*, glypican 3 (GPC3), keratin (KRT) 8, 18, and 19, paired box 1 and forkhead box 1, Noto, Brachyury, and glioma-associated oncogene (Gli) 1 and 3 [26, 27]. In the immature and young IVD, the NP also contains large vacuolated cells that are referred as notochordal cells, whose number gradually decreases with advancing age [28]. Considering the ratio between the cell number and the disc volume, the IVD is considered to be relatively acellular. Indeed, it presents an average cell density of 5.8 × 10^3^ cells/mm^3^ (with the NP containing 4 × 10^3^ cells/mm^3^ and the AF with 9 × 10^3^ cells/mm^3^); however, such values progressively decline with aging [29]. In the healthy IVD, cell anabolism and proliferation are orchestrated by numerous growth factors that are normally present in the disc. These include members of the transforming growth factor *β* (TGF-*β*) superfamily, insulin-like growth factor 1 (IGF-1), epidermal growth factor (EGF), connective tissue growth factor (CTGF), bone morphogenetic protein- (BMP-) 2, osteogenic protein-1 (OP-1), and growth and differentiation factor- (GDF-) 5 and 6 [30]. Several *in vitro* and *in vivo* studies have showed the capacity of such factors to enhance cell proliferation, proteoglycan, and collagen synthesis while reducing the production of metalloproteinases and proinflammatory cytokines [30].

Recent studies have demonstrated the existence of a population of progenitor stem cells within the IVD. These cells have been isolated from the NP, AF, CEP, and niches located in the perichondrium outside of the epiphyseal plate [12]. Such cells exhibited plastic adherence capacity, multilineage differentiation *in vitro*, and expression of a characteristic surface marker pattern (CD73^+^ CD90^+^ CD105^+^ CD11b^−^ CD14^−^ CD19^−^ CD34^−^ CD79^−^ HLADR^−^) and thus can be classified as NP-MSCs [31]. However, compared to bone marrow-derived MSCs (BM-MSCs), NP progenitor cells isolated from degenerated IVDs showed a reduced or even defective adipogenic differentiation [32]. In addition, NP-MSCs exhibited increased viability and proliferative capacity under hypoxic and acidic conditions compared to AD-MSCs *in vitro* [33, 34]. Furthermore, a specific subpopulation of clones derived from IVD progenitors expressing the tyrosine protein kinase receptor TIE2 and the disialoganglioside GD2 was recognized for its superior capacity to proliferate, form spheroid colonies, synthesize ECM components, self-renew, and differentiate into diverse lineages, both *in vitro* and *in vivo* [35]. More specifically, TIE2^+^ GD2^−^ cells are considered to be dormant precursors of TIE2^+^ GD2^+^ cells; this switch denotes the activation of NP progenitor cells and the irreversible and unidirectional differentiative process characterized by the loss of TIE2 and GD2 and the expression of CD24, eventually leading to the acquisition of the mature NP cell phenotype [31]. In this context, angiopoietin-1 (ANG-1), a ligand of the TIE2 receptor, has been shown to stimulate the formation of NP progenitor cell colonies *in vitro*. This molecule may thus be a key regulating factor in the IVD stem cell niche [35].

Due to its unique anatomical location and function, the IVD displays intrinsic physicochemical features that collectively establish a harsh microenvironment for both resident cells and transplanted exogenous cells, whose viability and functionality may thus be significantly blunted [36]. Indeed, the IVD presents with an avascular, nutrient-deficient, hypoxic, acidic, hyperosmolar, and mechanically solicited environment [37]. Such conditions even worsen with the onset and progression of IDD [38]. Main IVD microenvironment characteristics are summarized in Table 1.

## 3. Intervertebral Disc Degeneration: A Great Challenge for Cell Survival

The pathophysiology of IDD is complex and still not completely understood. Major contributors include aging [38], smoking [52], mechanical overload [53], obesity [54], and diabetes [55]. The structural hallmark of IDD is the progressive dehydration of the NP mainly due to the gradual loss of proteoglycans within the ECM [19, 56]. The principal proteolytic enzymes involved include the matrix metalloproteinases (MMPs) and a disintegrin and metalloproteinase with thrombospondin motifs (ADAMTS) [57] which are upregulated together with proinflammatory cytokines, including tumor necrosis factor *α* (TNF-*α*), interleukin- (IL-) 6, IL-1, IL-17, and interferon *γ* (IFN-*γ*) [51], while anabolic factors, such as IGF-1 [58] and TGF-*β* [59], appear to be downregulated. Tissue inflammation [51], increased oxidative stress [60], mechanical overload [61], and the prevalence of catabolic events notably impact on NPCy viability by boosting cell senescence [62] and apoptosis [55, 63].

Cell senescence is defined as the irreversible arrest of the cell cycle with loss of proliferative capacity, reduced anabolic activity, and aberrant production of metalloproteinases, proinflammatory cytokines, and chemokines [64]. During IDD, cell senescence is triggered by several stimuli, including oxidative stress, acidity, nutrient depletion, mechanical overloading, and tissue inflammation [65]. These lead to DNA damage and the activation of the p53-p21-Rb, p38-p16-Rb, and Wnt/*β*-catenin pathways, which ultimately cause the acquisition of the senescent phenotype [66]. As IDD progresses, the number of senescence-associated *β* galactosidase- (SA-*β*-Gal-) positive cells increases and positively correlates with the Thompson and Pfirrmann degeneration grade of degenerated IVDs [64].

The reduced number of functional cells progressively results in the incapacity of NPCy to counteract degenerative changes by producing an adequate amount of ECM to maintain IVD structural integrity [67]. Furthermore, the enhanced secretion of proinflammatory and catabolic factors by senescent cells may favour the senescence of neighbouring healthy cells thus precipitating a sort of degenerative domino effect. The strong relationships among IDD, cell senescence, and inflammation have been recently described as “inflammaging,” a process with distinctive biological and proteomic features typical of aging and degenerating IVDs [68].

Eventually, NP dehydration leads to a decrease of disc height and shock-absorbing properties of the NP itself, thus causing circumferential forces to be transmitted to the AF which eventually result in the formation of cracks and fissures, providing a basis for the development of discogenic LBP, disc herniations, and segmental instability [69].

### 3.1. Avascularity and Nutrient Deficiency

As stated above, the nourishment of IVD tissues strictly depends on the diffusion of nutrients from the capillaries running through the CEP [21]. Therefore, the IVD itself is inherently avascular. The diffusion process may be further hampered because of end plate calcification and subsequent capillary obliteration, which is common in IDD due to IVD aging and mechanical overstress [39]. The lack of blood flow consequently establishes a hypoxic microenvironment with higher O_2_ concentrations at the AF surface (19.5%) that then drop towards the center of the NP (0.65%), where cell survival is fully maintained even when oxygen tension falls below 5% [40]. Resulting oxygen concentration is determined by the balance among O_2_ transportation across the CEP, cell density, and metabolism [70]. As a consequence of avascularity, IVD tissue are exposed to low glucose concentrations that vary from approximately 5 mM at the periphery to circa 0.8 mM in the center of the IVD [42]. To maintain their viability, IVD cells adjust their metabolic requirements to the hypoxic low glucose environment by mainly relying on anaerobic glycolysis, which allows generating energy while consuming less O_2_ and producing less reactive oxygen species (ROS) [71]. Such degree of adaptation has been confirmed by the fact that NPCy are able to survive in hypoxic conditions by HIF-1 and -2 expression, which positively regulate cell proliferation, energy metabolism, type 2 collagen production, oxidative stress, and cell autophagy and apoptosis. These mechanisms may hold a fundamental role in the adaptation of NPCy to even lower levels of oxygen and nutrients as it occurs during IDD [72]. Similarly, BM-MSCs and adipose-derived stem cells (ADSCs) have been demonstrated to increase cell viability and synthesis of ECM components in hypoxic (1-5% O_2_) and low glucose conditions *in vitro* [36, 73]. BM-MSCs have shown a greater development of colony-forming units (CFU) [74], a decrease of osteogenic differentiation [75], and an attenuation of the inhibitory effect of IL-1*β* towards chondrogenic differentiation when exposed to a hypoxic environment [76]. Moreover, hypoxia has demonstrated to inhibit MSC senescence and to maintain cell stemness through stimulation of telomerase activity via the HIF1*α*-Twist-mediated downregulation of the E2A-p21 pathway [77]. Collectively, these adaptive strategies may favour MSC survival in the hostile IVD microenvironment after implantation. However, it has been shown that the prolonged exposure to severe hypoxia (oxygen tension < 1%) together with serum deprivation eventually resulted in complete cell death [78].

MSC viability and differentiation into a NPCy-like phenotype may be further enhanced by the supplementation of growth factors, namely, TGF-*β*, IGF-1, growth differentiation factor- (GDF-) 5, GDF-6, platelet-derived growth factor (PDGF), and basic fibroblast-like growth factor (bFGF). Such molecules are normally expressed by IVD resident cells, but as cell density decreases, nutrient diffusion hinders and ECM becomes disrupted; their anabolic effect gets almost completely blunted. For this reason, preexposing MSCs *in vitro* to such growth factors before implantation or embedding them in MSC-loaded bioscaffolds could maintain their biological activity and boost cell proliferation, differentiation, and synthetic potential [37]. Several *in vitro* and preclinical studies have been conducted in this regard [79–83].

### 3.2. Acidity

Due to the extensive recourse to anaerobic glycolysis by disc cells, lactic acid is produced and accumulated within IVD tissues (average concentration between 2 mM and 6 mM), whose average pH is slightly acidic (7.0-7.2) in physiological conditions. However, in mild degenerative conditions, the pH may drop to 6.5 and even to 5.6 in severely degenerated IVDs, with a significant effect on NP cell viability and ECM composition resulting in a reduction of collagen and proteoglycan synthesis [38]. pH variations are recognized by IVD cells through acid-sensing ion channels (ASICs), which regulate Ca^2+^ transmembrane influx upon fluctuations of extracellular H^+^ levels. Moreover, ASIC expression by both NPCy and AF cells is upregulated in IDD, thus suggesting the crucial role of these channels [84]. Human IVD cells express all ASIC isoforms (ASIC-1, ASIC-2, ASIC-3, and ASIC-4); ASIC-1a activation has been shown to increase Ca^2+^ cellular influx eventually resulting in apoptosis of CEP chondrocytes [85], while ASIC-3 has been involved in NPCy survival under acidic and hyperosmolar conditions *via* the nerve growth factor (NGF)/p75/extracellular signal-related kinases (ERK) pathway [86]. However, NGF is also renowned for its role in IDD as it promotes nerve ingrowth within the injured IVD and hence favours the development of discogenic LBP [87]. Based upon these observations, it has been questioned whether the survival of disc cells within the acidic environment may be enhanced by inhibiting ASIC1a and/or stimulating ASIC3, although the increased expression of the latter in dorsal root ganglions has been associated with enhanced radicular pain [88]. Gilbert et al. elegantly showed that, when exposed to a pH of 6.8 *in vitro*, human NPCy viability remained high while proliferation stopped. At lower pH values, cells began to die and secrete significant amounts of proinflammatory cytokines (IL-1*β* and ΙL-6) and neurotrophic pain-related factors, including NGF and brain-derived neurotrophic factor (BDNF). Highly acidic culture conditions also resulted in a decrease of aggrecan content together with an upregulation of MMP-3, ADAMTS-4, and ASIC-3, while ASIC-1 and ASIC-2 levels remained unchanged. Interestingly, when ASIC-3 was selectively inhibited using APETx2, the increase of cytokines and neurotrophic factors was prevented, even if no positive effect was reported on cell viability nor on ECM breakdown in acidic conditions. However, this study pointed out again the importance of ASIC-3 in triggering the pH-dependent inflammatory and neurogenic response of NPCy [89].

Differently from IVD cells, MSC biosynthetic activity is largely impaired when exposed to acidic pH *in vitro*. A study from Wuertz et al. showed that rat BM-MSCs underwent a significant decrease of cell viability and proliferation together with the inhibition of aggrecan, type 1 collagen, and tissue inhibitor of metalloproteinases- (TIMP-) 3 even when exposed to a pH corresponding to a mild degenerative condition (6.8) [90]. More recently, Liu and colleagues reported that, with decreasing pH (from 7.4 to 6.2), human NP-MSCs displayed a reduction of cell proliferation, an increase of apoptosis, and a diminished gene expression of type 1 collagen, type II collagen, aggrecan, and SOX-9, as well as of stemness-related genes (Oct4, Nanog, Jagged, and Notch1) *in vitro*. Contrariwise, as pH decreased, ASIC-1, ASIC-2, ASIC-3, and ASIC-4 expression gradually increased. Blocking ASIC activity by using amiloride resulted in a significant improvement of cell viability and a normalization of ECM and stem cell-related gene expression, thus suggesting the importance of ASICs in the negative regulation of IVD cell metabolism within the acidic degenerative disc environment [91]. Similar results were previously documented by Han et al., who compared the effect of the acidic environment on AD-MSCs and NP-MSCs eventually reporting that the latter were less sensitive to the inhibitory and catabolic consequences of low pH, thus being more promising as candidates for IVD regeneration [34].

### 3.3. Hyperosmolarity

Due to the large amount of negatively charged GAG side chains of aggrecan molecules, the IVD and especially the NP are characterized by a high osmolarity, which is responsible for the notable imbibition and swelling pressure of the NP in physiological conditions. However, NP osmolarity (and thus water content) is not fixed but cyclically varies upon applied mechanical load; indeed, it diminishes by 20-25% during diurnal activities while it is restored at rest, with values ranging between 430 and 500 mOsm/L *in vivo* [44]. Due to the progressive loss of proteoglycans with IDD, IVD osmolarity declines as the degenerative process goes on [45].

NPCy are able to sense changes of osmolarity by regulating the expression of tonicity enhancer-binding protein (TonEBP), which is activated in hypertonic conditions and binds to the tonicity-responsive enhancer element (TonE) motif, eventually resulting in the upregulation of downstream genes, namely, heat shock protein 70, betaine/*γ*-aminobutyric acid transporter, sodium myo-inositol transporter, taurine transporter, and aquaporin-2, which are fundamental to control intracellular osmotic stress [92, 93]. However, hyperosmolarity was not found to induce autophagy in NPCy as it occurs with other cell types [94]. Moreover, TonEBP activation may positively regulate the production of ECM components by directly increasing the expression of both aggrecan and galactose-*β*1,3-glucuronosyltransferase-I (GlcAT-I), which is a key enzyme for the synthesis of GAG side chains [95]. Notwithstanding, a recent *in vitro* study showed that although hyperosmolarity upregulated ECM gene expression, no notable increase was noted at the protein level [96]. TonEBP is also involved in the upregulation of proinflammatory cytokines under hypertonic stress, including C-C motif chemokine ligand (CCL), nitric oxide synthase 2 (NOS-2), IL-6, and TNF*α* [97].

An excessive hypertonic stress has been documented to induce DNA damage and subsequent arrest of the cell cycle in bovine NPCy, though cells exhibited the capacity to repair DNA after the genotoxic insult [98]. An interesting study conducted by Li and colleagues using a whole organ-cultured porcine IVD model assessed the effect of hypoosmolarity (330 mOsm/L), isoosmolarity (430 mOsm/L), hyperosmolarity (550 mOsm/L), and cyclic osmolarity (430 mOsm/L for 8 h and then 550 mOsm/L for 16 h) on NPCy viability and ECM synthesis. They found that hypo- and hyperosmolarity significantly increased NPCy apoptosis compared to isoosmolarity and cyclic osmolarity; the process was even more pronounced after the inhibition of ERK 1/2, which may physiologically protect NPCy against osmotic stress-induced apoptosis [99]. Similarly, expression and protein synthesis of SOX9, aggrecan, and type 2 collagen as well as GAG content were notably higher in isoosmolarity and cyclic osmolarity cultures [100].

Mizuno et al. investigated the effect of different combinations of hydrostatic and osmotic pressures on bovine NPCy *in vitro* and demonstrated that the association of a cyclic hydrostatic stress (0.5 MPa, 0.5 Hz) with a high osmolarity (450 mOsm/L) mimicking daily spinal stress resulted in an upregulation of aggrecan, type II collagen, and other anabolic markers [101].

Differently from mature NPCy, notochordal cells can better adapt to fluctuations in osmolarity. In case of a hypotonic stress, notochordal cells have shown to release a low osmotic solution from intracellular vacuoles so as to dilute the cytoplasm and maintain an osmotic equilibrium across the membrane thus preventing excessive cell swelling and death [102]. Conversely, hyperosmolarity has demonstrated to induce notochordal cell differentiation towards a mature NP cell phenotype characterized by upregulation of aquaporin-3 and downregulation of N-cadherin [103].

Less is known about the effect of hyperosmolarity on MSCs. A previous study has shown that hypertonic culture conditions reduced BM-MSC proliferation, anabolic marker expression, and chondrogenic differentiation [73]; similarly, AD-MSC viability and proliferation together with aggrecan and type 1 collagen expression were abated [36]. In a recent study, Li et al. showed that exposing NP-MSCs to osmotic pressures mimicking the healthy IVD environment (430-500 mOsm/L) resulted in a decrease of cell proliferation and chondrogenic differentiation *via* activation of the ERK pathway, while the relative hypoosmotic condition of mild IDD proved to increase NP-MSC proliferation and chondrogenic potential. Based on this study, a degenerative microenvironment, due to lower osmotic pressures, may be less hostile to NP-MSCs, providing an interesting insight for future regenerative strategies [104].

### 3.4. Mechanical Loading

IVDs, especially in the cervical and lumbar regions, are naturally exposed to complex biomechanical stimuli, including flexion, torsion, shear, and compression. Diverse load intensity, duration, frequency, and direction profoundly influence IVD cell activity and ECM dynamic composition [46]. Normally, intradiscal pressure (L4-L5) is approximately 0.1-0.24 MPa in supine position and may increase up to 2.0 MPa when carrying a 20 kg weight with the flexed back [47]. It has been previously shown that, at physiological pressures, MMP-3 production is reduced while TIMP-1 synthesis is increased, thus exerting a net anabolic effect on the ECM. Conversely, as intradiscal pressure drops during IDD due to NP dehydration, the MMP-3/TIMP-1 ratio is inverted and catabolic events predominate. In addition, IVD cells harvested from degenerated discs seem to exhibit a less anabolic response at a physiological intradiscal pressure [46]. When exposed to increasing mechanical loads, NPCy get overstressed due to mitochondrial damage and excessive ROS production. Under a certain threshold, NPCy can counteract such stress by activating autophagy and removing damaged mitochondria so as to reduce the generation of ROS [105]. However, with prolonged loading, NPCy irreversibly undergo apoptosis through caspase-dependent mitochondrial pathways. In addition, a specific form of programmed cell death with necrosis-like features, namely, necroptosis, seems to be involved in the compression-induced death of NPCy [106].

The effect of different loading modalities on IVD cells has been extensively investigated by several studies. In most investigations, static compressive loading has been associated with increased cell death and matrix catabolism due to diminished expression of collagen and aggrecan while metalloproteases were produced in higher amounts. Conversely, dynamic compressive loading has been described to often elicit an anabolic response and to enhance transportation of nutrients and especially high molecular weight molecules, such as growth factors (IGF-1, TGF-*β*, FGF, and PDGF), within the NP [48]. The type, degree, and duration of metabolic responses of IVD cells to loading strictly depend upon experimental design, cell source, age of donor, and culturing conditions and have been reviewed elsewhere [46, 48].

Similarly to IVD cells, cyclic mechanical loading has been shown to stimulate ECM production and chondrogenic differentiation of BM-MSCs [26] and NP-MSCs [37] *in vitro*. A study from Gan et al. demonstrated that the anabolic response of BM-MSCs to low-magnitude compression was triggered by the activation of the mechanotransducer transient receptor potential vanilloid 4 (TRPV-4) on the cell surface [107]. While mechanical loading may be a convenient resource to predifferentiate MSCs in view of a subsequent cell therapy, it could be speculated that the enhanced chondrogenic commitment of NP progenitors upon prolonged mechanical stimulation may contribute to the exhaustion of the resident stem cell compartment during IDD. This has been also demonstrated by previous studies showing an increased NP-like differentiation upon dynamic loading of AD-MSCs [108] and the acquisition of an AF-like phenotype by BM-MSCs when exposed to radial compressive stimulation [109]. Differently from dynamic loading, static prolonged loading (1 MPa, >24 h) significantly diminished NP-MSC viability, migration, differentiation, CFU formation, and stemness-related gene expression, suggesting that biomechanical overload might be a cause of endogenous repair failure for IVD regeneration [110]. However, in a coculture experiment involving AD-MSCs and NPCy undergoing high prolonged loading (3 MPa for 48 h), the presence of AD-MSCs resulted in a reduction on NPCy apoptosis *via* the inhibition of activated caspase-9 and 3 as well as in an upregulation of ECM genes with diminished expression of metalloproteinases and proinflammatory cytokines (IL-1*β*, IL-6, and TNF-*α*) [111].

### 3.5. Inflammation

IDD is thought to be sustained in part by the abnormal secretion of proinflammatory cytokines by AF cells, NPCy as well as by T cells, neutrophils, and macrophages located within the IVD. A wide array of inflammatory mediators is involved in the process, including interleukins (IL-1, IL-2, IL-4, IL-6, IL-8, IL-10, and IL-17), TNF-*α*, IFN-*γ*, chemokines, and prostaglandin E_2_ (PGE_2_). These molecules are able to induce cell apoptosis, senescence, and autophagy, to favour the development of discogenic LBP and to upregulate the synthesis of MMPs (-1, -3, -7, -9, and -13) and ADAMTS (-1, -4, -5, -9, and -15) by NPCy, thus leading to ECM breakdown [51].

IL-1*β* is considered a key molecule in the IDD-driven inflammatory response. Indeed, IL-1*β* expression is positively associated with the severity of IDD and promotes ECM catabolism both by upregulating proteolytic enzymes and inhibiting aggrecan expression and synthesis [112]. In addition, it has been shown that IL-1*β* can reduce aggrecan and SOX9 expression by rat NP-MSCs and improve their neurogenic potential, which may eventually result in IVD neoinnervation and generation of LBP [49]. However, it has been noted that IL-1*β* is expressed even in healthy IVDs since birth, thence possibly presenting an unknown physiological function. Interestingly, a recent study from Gorth et al. reported that, compared to controls, IL-1*α*/*β* knockout mice unexpectedly showed bone morphology alterations and higher degrees of IDD mainly involving the AF, while NP structure remained mostly unaffected [50].

Together with IL-1*β*, TNF-*α* holds a fundamental role in IVD inflammation as the two cytokines are present at high levels in degenerated and herniated disc as well as in the epidural space [51]. Furthermore, TNF-*α* is able to amplify the inflammatory response by stimulating IVD cells to synthesize additional cytokines, including IL-6, IL-8, IL-17, IL-1*β*, and substance P (SP), and numerous chemokines. Among the latter, CCL-5 has been shown to attract eosinophils and macrophages towards the inflamed IVD and to correlate with both discogenic LBP and the severity of IDD [113].

A previous study has reported that MSCs are able to secrete anti-inflammatory mediators (IL-1 receptor antagonist, IL-10, IL-13, and tumor necrosis factor-inducible gene 6 protein), anticatabolic factors (TIMPs), and growth factors (TGF-*β*, GDF-5) when cultured in IDD-like conditions, thus exerting an immunomodulatory effect on surrounding cells [114]. In a recent study, Teixeira and colleagues evaluated the biological responses of human BM-MSCs using an *ex vivo* bovine model of IDD (needle puncture with IL-1*β* supplementation). While not presenting a tangible effect on ECM production, BM-MSCs showed an increased production of IL-6, IL-8, PGE_2_, and monocyte chemoattractant protein 1 (MCP-1) and a downregulation of the expression of IL-6, IL-8, and TNF-*α* by bovine IVD cells [79]. Moreover, MSC migration to inflamed IVD tissues was enhanced probably due to the presence of IL-1*β*, which has renowned chemoattractant properties towards MSCs [115]. Indeed, many of the proinflammatory mediators that have been previously cited exhibit chemotactic features which may be crucial in the recruitment of MSCs (both endogenous and exogenous) into the degenerating IVD by resident NP cells [106]. On the other hand, MSCs have been described to secrete cytokines and factors that support NP cell viability and functionality under stress. For example, TGF-*β* and IGF-1 have shown to inhibit NP cell senescence [116], while IGF-1 and bone morphogenetic protein 7 protected NPCy against apoptosis [91] and together with TGF-*β* reduced ECM degradation and inflammation [117].

## 4. Intervertebral Disc Regeneration: The Need to Learn from the Disc Microenvironment

**Figure 1:**
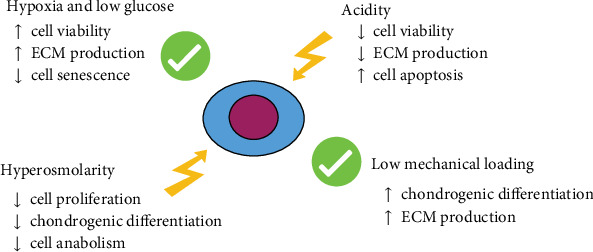
Schematic representation of the major effect of the IVD microenvironment on MSCs based upon actual evidences. ECM = extracellular matrix.

As IDD is mainly sustained by progressive NPCy depletion, several research efforts have been conducted to develop biological treatments that may replenish the NP with active and functional cells. In the last decades, numerous attempts to regenerate the IVD by introducing viable stem cells into the degenerating disc have been made, using different cell sources, animal models, injection routes, and bioengineered scaffolds [12, 118]. Among the diverse cell utilized in the studies within the field, MSCs have been widely adopted for their prompt availability (as they can be easily and safely harvested from the bone marrow and the adipose tissue) and the capacity to differentiate along the chondrogenic pathway [9]. In addition, MSCs acquire a NPCy-like phenotype when exposed to specific growth factors *in vitro* [119] and have been shown to increase ECM production and even to improve radiographically assessed disc height and IVD hydration at T2-weighed magnetic resonance imaging (MRI) in different preclinical studies [31, 120]. The rationale behind intradiscal cell therapy is to increase NP cellularity due to the differentiation of MSCs into functional NPCy and to support residual NPCy activity through the secretion of growth factors, anti-inflammatory cytokines, and anticatabolic mediators, thus eventually resulting in ECM restoration and regeneration of the IVD [106]. Furthermore, the recent individuation of endogenous IVD stem/progenitor cells has raised the possibility that this mechanism may naturally occur during IDD upon release of specific cues by resident IVD cells that could recruit local progenitors from stem cell niches to regenerate the tissues. However, as IDD advances, both progenitor cell number and ability to migrate (and hence their intrinsic regenerative capacity) decline because of the hostility of the degenerating IVD microenvironment [106]. The lack of a full regenerative response and, in some studies, the absence of significant changes compared to the controls, apart from methodological issues, have been imputed to the unlikely capacity of transplanted cells to survive in the degenerating IVD harsh microenvironment, which is profoundly different from stem cell niches [37]. While hypoxia [76] and mechanical loading [26] have been described to enhance MSC differentiation, proliferation, and anabolism, acidic pH [90, 91] and hyperosmolarity [73] drastically impair cell viability and phenotype (Figure 1).

In addition, the cell response to the degenerating microenvironment may significantly vary when comparing NP-MSCs, BM-MSCs, and ADSCs (Table 2). Among the three cell types, NP-MSCs tend to exhibit a higher chondrogenic differentiation capacity, considering that MSCs isolated from a specific tissue are more prone to acquire the residing cell phenotype [121]. However, the role of NP-MSCs in IDD cell therapy may be significantly limited by the low number of available cells, by the reduced regenerative potential due to the degree of IDD at the time of cell harvest and by the need of an invasive approach to the IVD for NP-MSC retrieval [33]. Conversely, BM-MSCs have been demonstrated to be promptly available, easy, and safe to harvest and able to maintain a significantly high viability and CFU capacity even in adverse conditions. This cell type is the most extensively investigated with regard to IVD regeneration and thus constitute the most convenient source for intradiscal cell therapy at the present time [9]. ADSCs have also shown to hold potential for regenerating the IVD. Even with a lower ability to differentiate along the chondrogenic line and a reduced capacity to tolerate the acidic and hyperosmolar microenvironment compared to NP- and BM-MSCs, the use of ADSCs may be favoured by their high proliferation rate and the little donor site morbidity [36].

In the last years, the growing body of preclinical research has confirmed the safety, feasibility, and efficacy of MSC-based intradiscal cell therapy, thus funding the basis for clinical application. Orozco et al. conducted a pilot study on 10 patients affected by LBP unresponsive to conservative treatment. Patients were injected with autologous expanded BM-MSCs into the NP area; pain and disability (measured with the Visual Analogue Scale (VAS) and Oswestry Disability Index (ODI), respectively) were significantly reduced at 3, 6, and 12 after injection. In addition, although disc height was not restored, MRI demonstrated a significant elevation of NP water content at 12 months [122]. Similar results were described by Pettine et al., who reported that 8 of 20 treated patients showed a reduction of one modified Pfirrmann grade at 12 months. Furthermore, improvement of pain and disability was faster in patient receiving a higher dose of BM-MSCs but reduced in patients >40 years of age, suggesting that MSC regenerative capacity may depend on both cell concentration and patients' characteristics [123]. The study by Centeno et al. was conducted on 33 patients affected by LBP and radiculopathy and injected with autologous BM-MSCs. The follow-up period was extended until 6 years; pain was significantly reduced at 3, 36, 48, 60, and 72 months after injection, as well as reported functionality (functional rating index, FRI), which was improved at each time point excluding 12 months. In addition, 17 patients showed a reduction of the disc herniation volume (average reduction of 23% in size) [124]. The first, small randomized controlled trial has been conducted by Noriega et al., who randomized 24 patients with degenerative LBP treated with either sham infiltration or allogeneic BM-MSCs from healthy donors.

40% of the patients in the experimental group showed a rapid improvement of pain and functionality as well as Pfirrmann grading, which instead worsened in the control group [126].

The only clinical evidence of an intradiscal therapy involving ADSCs has been provided by Kumar et al., who performed a phase I clinical trial on 10 patients affected by chronic LBP. Patients were injected with a combination of hyaluronic acid (HA) and ADSCs and followed up to 1 year. 60% of individuals displayed a significant improvement of pain, disability, and quality of life, with 3 patients additionally showing increased water content as demonstrated by MRI [127].

Overall, intradiscal cell therapy has demonstrated to be safe in all previous reports; no major adverse events were observed, while minor event basically consisted in local pain treated with analgesics [128].

Another strategy that is being widely investigated in the field is tissue engineering, whose principal aim is to mimic the natural IVD microenvironment through the combination of biomaterials, soluble factors, and functional cells so as to reproduce IVD original biological and biomechanical properties. Regarding IDD, three main bioengineering approaches have been developed: repair of the AF, NP regeneration or replacement, and NP/AF combined repair [25].

AF fissures and tears usually occur with IDD due to higher tensile and compressive stresses transmitted to the AF as a consequence of NP dehydration and loss of disc height. At the latest stages, AF disruption may lead to NP displacement thus causing IVD herniation [129]. In this regard, a strategy to repair the AF may result in the restoration of IVD function and prevention of reherniation. Different suturing devices and polymeric meshes have been employed to close the annular defect; such approaches have demonstrated to be efficacious and safe in numerous *in vitro* studies [130] and in some clinical applications, although the complexity of the techniques and their consistent costs have limited their diffusion [25]. Alternatively, diverse biomaterials have been exploited as void fillers of the AF damage in the form of hydrogels and sponges (polylactic acid, polyglycolic acid, GAGs, poly(s-caprolactone), silk, etc.) with or without a cellular component. Indeed, BM-MSCs engrafted within a poly(trimethylene carbonate) scaffold and covered by a poly(ester-urethane) membrane were tested in a bovine organ culture annulotomy model. After 14 days under dynamic load, disc height was restored and no reherniation occurred. Moreover, MSCs displayed an increased expression of anabolic genes and markers of the AF phenotype [131].

Tissue engineering attempts to regenerate the NP ideally aimed at reproducing the nucleus functions so as to restore disc height and spinal biomechanics. Hydrating synthetic polymers were firstly investigated for their potential ability to mimic the water retention capacity of GAGs lost during IDD thus leading to increased IVD height and pressure. However, the *in vivo* application of such materials often resulted in excessive swelling with consequent development of segmental stiffness, end plate fractures, and device failure [25]. A similar strategy that is gaining growing interest is the design of synthetic polymers that undergo a transition from an easily injectable form to a gel-like form when exposed to particular pH levels or when combined with cross-linking molecules [132]. Biomaterials that have been tested in this field include HA, GAG-containing scaffolds, and collagen. Again, this approach mainly focuses on biomechanical goals and the integration and interaction of the implants with resident cells have been neglected in most studies [25]. The most appealing solution to regenerate the NP at the present time seems to be the design of proper scaffolds that could provide transplanted MSCs with an adequate three-dimensional microenvironment. The ideal scaffold should be biocompatible and easy to manipulate, while biomechanically stable and respectful to cell morphology and functionality [133]. To date, various biomaterials including HA, collagen, fibrin, alginate, gelatin, and silk have been investigated as scaffolds for cell engraftment and IVD regeneration [12]. Although there is no definitive evidence for preferring a carrier than another, hydrogels are being more thoroughly evaluated due to the high-water content (90-95%) similar to the healthy NP ECM and their easy handling and tunability. Hydrogels are composed of natural or synthetic macromolecules that can be assembled to be either “mechanically competent” or “biologically competent” [134]. The former are constituted by highly cross-linked molecular networks that are able to increase disc height and withstand biomechanical stimuli, while not allowing for an adequate matrix hydration and cell encapsulation. On the other hand, “biologically competent” hydrogels present a less cross-linking degree which permits embedded cells to proliferate, differentiate, and produce ECM components whereas they lack adequate biomechanical characteristics [135]. Major drawbacks of these biomaterials have recently been overcome by the development of double network hydrogels, which combine a highly cross-linked network (providing the scaffold with mechanical competence) with a less dense network that may consent cell survival and matrix deposition, and thus hold a promising role in IVD regeneration [136]. Recent studies have reported that the macromolecules employed to build hydrogels for IVD regeneration may modulate the harsh degenerative microenvironment by naturally presenting anti-inflammatory properties or by being combined with other active molecules. Indeed, Teixeira et al. have showed that the injection of chitosan/diclofenac/*γ*-polyglycolic acid nanoparticles was able to reduce the production of IL-6, IL-8, and PGE_2_ in an organ model of IDD [137]. HA is a high molecular weight-nonsulfated GAG which is normally expressed within the healthy IVD matrix. Such molecule is fundamental in multiple biological processes, including cell migration, survival, apoptosis, and morphogenesis as well as tumorigenesis and tissue inflammation [138]. For these reasons, HA is being widely investigated as a bioscaffold in IVD regeneration in various formulations. A recent *in vivo* study demonstrated that the intradiscal injection of HA in a rat model of IDD resulted in a strong analgesic effect with the reduction of hyperalgesia, allodynia, and sensory hyperinnervation, while decreasing the expression of IL-1*β* and IL-6 and the deposition of fibrous tissue within the ECM [139].

In addition, biomaterials can be further implemented with biological cues that may boost differentiation and anabolism of the cells embedded in the scaffold. For example, the combination of TGF-*β* with a MSC-embedded polymerized fibrin scaffold implanted in a rat model demonstrated to inhibit MSC apoptosis and led to an increased disc height when compared to the scaffold alone [16]. Similarly, in another study, BM-MSCs were cultured with collagen microcarriers functionalized with either TGF-*β* or bFGF; while the former was significantly upregulated MSC chondrogenic differentiation and production of aggrecan, collagen, and proteoglycan, bFGF notably increased cell proliferation [140].

An additional innovative solution involves the use of self-assembling peptides that are able to form stable nanofiber hydrogels which can be further enriched with specific motifs that have been shown to improve cell migration, adhesion, proliferation, and differentiation [141].

## 5. Conclusions

The IVD is a complex organ with unique physicochemical characteristics; the absence of vasculature, hypoxia, low glucose concentration, acidity, hyperosmolarity, and continuous mechanical stimulation contribute to establish a hostile microenvironment for cell survival and endogenous tissue repair. With the progression of IDD, such asperities become harsher and are further worsened by the local inflammatory response, eventually leading to excessive resident cell death, ECM breakdown, and loss of IVD original features. Intradiscal cell therapy has raised the possibility to regenerate the IVD by restoring the functional cell compartment and reversing the degenerative changes of IDD. However, the design of an efficacious and long-lasting therapeutical approach must carefully consider the deleterious effects of the IVD microenvironment on implanted cells. In this regard, more efforts are needed to better identify the most suitable cell source and an adequate scaffold that may provide an immediate mechanical support while allowing for cell nourishment, proliferation, differentiation, and synthesis of anti-inflammatory and trophic factors. As our understanding of IDD is still limited, it is essential to conduct further studies to better comprehend how the IVD microenvironment orchestrates local biology and how it impacts on exogenously delivered cells when far from their original niche.

## Figures and Tables

**Table 1:** Main IVD microenvironment features under physiological and degenerative conditions.

	Healthy IVD	IDD
Avascularity	Vessels from the vertebral bodies branch into capillaries terminating in the CEP [21]	CEP calcification may hinder nutrient diffusion [39]
Hypoxia	Oxygen concentration decreases from AF surfaces (19.5%) to the inner portion of the NP (0.65%) [40]	Oxygen concentration falls due to reduced blood supply and shift of NP cell metabolism towards oxidative phosphorylation [41]
Low glucose concentration	Glucose concentration is higher at IVD boundaries while it falls towards the center of the NP [42]	Glucose levels diminish together with blood supply and increased consumption by degenerative cells [43]
Acidity	Due to anaerobic glycolysis and lactic acid production, average pH is 7.0-7.2 [38]	pH may decrease to 6.5 in mild IDD and 5.6 in severe IDD due to nutrient depletion and increase lactic acid production [38]
Hyperosmolarity	The high GAG content within the NP determines a high osmolarity which varies upon mechanical load (430-500 mOsm/L) [44]	The loss of proteoglycans due to matrix breakdown reduces IVD osmolarity [45]
Mechanical loading	Mechanical stimuli (flexion, torsion, shear, and compression) regulate IVD cell activity and metabolism within a physiological range (0.1-2.5 MPa) [46, 47]	Disruption of IVD structure alters loading transmission across the IVD and the vertebral segments, resulting in tissue damage and cellular overstress [48]
Inflammation	Proinflammatory cytokines and chemokines may have a role in IVD development and recruitment of local progenitor cells [49, 50]	The excess of proinflammatory cytokines increases cell apoptosis, senescence, autophagy, matrix breakdown, and discogenic LBP [51]

CEP = cartilaginous end plate; AF = annulus fibrosus; NP = nucleus pulposus; IVD = intervertebral disc; IDD = intervertebral disc degeneration; GAG = glycosaminoglycan; LBP = low back pain.

**Table 2:** Different responses of NPCy, NP-MSCs, BM-MSCs, and ADSCs to the degenerative microenvironment.

	NPCy	NP-MSCs	BM-MSCs	ADSCs
Hypoxia and low glucose concentration	NPCy survive by relying on anaerobic glycolysis [71]. Low O_2_ concentration increases the expression of HIF-1 and 2, which upregulate cell proliferation and matrix production [72].	Low O_2_ concentration is associated with a higher viability and proliferative capacity of NP-MSCs compared to ADSCs [33, 34].	Hypoxia increases BM-MSCs CFU, reduces cell senescence and maintains cell stemness [74–77].	Low glucose slightly increases cell apoptosis and inhibits cell proliferation while enhancing aggrecan production [36, 73].
Acidity	NPCy survival at low pH is mediated by ASICs [84–86], even if critically acidic pH has been associated with decreased cell viability and upregulation of metalloproteinases and proinflammatory cytokines [87–89].	Low pH leads to reduced cell proliferation, enhanced apoptosis, and diminished expression of stemness-related and ECM genes [91]. However, overall performance was better than BM-MSCs and ADSCs [34].	Acidic pH significantly decreases cell proliferation, aggrecan, and type I collagen production [90].	Low pH promoted cell necrosis, reduced the proliferation rate, and diminished aggrecan production, while increasing type I collagen synthesis [36].
Hyperosmolarity	NPCy respond to hyperosmolarity through TonEBP activation [92, 93], which increases ECM gene expression [96]. However, excessive hyperosmolarity results in upregulation of proinflammatory cytokines and cell apoptosis [97–100].	Hyperosmolarity has been demonstrated to induce progenitor cell differentiation towards a mature NP phenotype [104].	Hypertonic conditions reduced BM-MSC proliferation, anabolism, and chondrogenic differentiation [73].	IVD-like hyperosmolarity significantly reduced ADSC viability and proliferative capacity and abated aggrecan and type I collagen synthesis [36].
Mechanical loading	Physiological loadings promote cell anabolism while abnormal mechanical stimuli cause ECM breakdown and reduced cell viability [46, 105].	Cyclic mechanical loading favours the differentiation of NP-MSCs towards mature NPCy [37], while static prolonged loading diminished cell viability, migration, differentiation, and stemness [110].	Cyclic mechanical loading enhances BM-MSC chondrogenic differentiation and cell anabolism [26].	ADCs may protect NPCy from apoptosis and promote the synthesis of ECM genes under prolonged loading [111].
Inflammation	Proinflammatory cytokines induce NPCy apoptosis, senescence, and autophagy and upregulate the synthesis of metalloproteinases, thus resulting in ECM breakdown [51, 112].	IL-1*β* may reduce aggrecan and SOX expression by NP-MSCs while improving their neurogenic differentiation, which may have a role in IVD neoinnervation [49].	BM-MSCs may support resident cells by secreting anti-inflammatory cytokines, anticatabolic, and growth factors [79, 114].	Under inflammatory conditions, ADSCs have been shown to increase proliferation, proinflammatory cytokine production, and osteogenic differentiation [125].

NPCy = nucleopulpocytes; NP-MSCs = nucleus pulposus-derived mesenchymal stem cells; BM-MSCs = bone marrow-derived mesenchymal stem cells; ADSCs = adipose tissue-derived mesenchymal stem cells; HIF = hypoxia-inducible factor; CFU = colony-forming units; ASIC = acid-sensing ion channels; ECM = extracellular matrix; TonEBP = tonicity enhancer-binding protein; NP = nucleus pulposus; IVD = intervertebral disc; IL = interleukin.
